# Fatty Acid Oxidation and Cardiovascular Risk during Menopause: A Mitochondrial Connection?

**DOI:** 10.1155/2012/365798

**Published:** 2012-02-01

**Authors:** Paulo J. Oliveira, Rui A. Carvalho, Piero Portincasa, Leonilde Bonfrate, Vilma A. Sardao

**Affiliations:** ^1^CNC—Center for Neuroscience and Cell Biology, University of Coimbra, 3004-517 Coimbra, Portugal; ^2^Department of Life Sciences, University of Coimbra, 3004-517 Coimbra, Portugal; ^3^Department of Internal Medicine and Public Medicine, Clinica Medica “A. Murri”, University of Bari Medical School, 70124 Bari, Italy

## Abstract

Menopause is a consequence of the normal aging process in women. This fact implies that the physiological and biochemical alterations resulting from menopause often blur with those from the aging process. It is thought that menopause in women presents a higher risk for cardiovascular disease although the precise mechanism is still under discussion. The postmenopause lipid profile is clearly altered, which can present a risk factor for cardiovascular disease. Due to the role of mitochondria in fatty acid oxidation, alterations of the lipid profile in the menopausal women will also influence mitochondrial fatty acid oxidation fluxes in several organs. In this paper, we propose that alterations of mitochondrial bioenergetics in the heart, consequence from normal aging and/or from the menopausal process, result in decreased fatty acid oxidation and accumulation of fatty acid intermediates in the cardiomyocyte cytosol, resulting in lipotoxicity and increasing the cardiovascular risk in the menopausal women.

## 1. Menopause: A Burden for Aging Women

**Figure 1:**
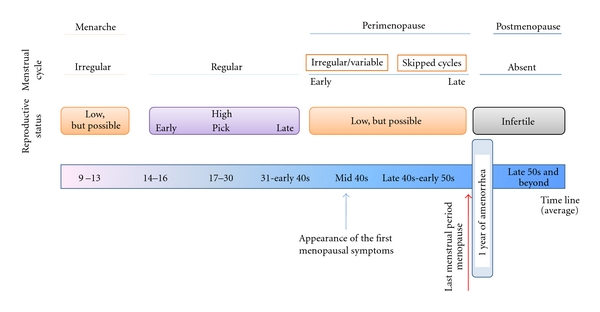
Women reproductive stages during aging: from menarche to postmenopausal. Time line represents only an average for the normal age. More details can be found in the text.

Menopause is one of the most critical periods in women's life. Although being a natural biological process that occurs with aging, physiological alterations observed during this period can be challenging. Caused by a reduced secretion of ovarian hormones estrogen and progesterone after depletion of the storage of ovarian follicles, menopause defines the end of women menstrual cycle and their natural fertility. On average, spontaneous or natural menopause occurs around the early 50s and is confirmed after 12 months of nonpathological amenorrhoea. However, when premature ovarian failure (POF) occurs before the 40s due to pathological causes, an early or premature menopause can be induced, which is thus disconnected from the aging process properly said. When a bilateral oophorectomy is necessary, menopause occurs immediately without women experiencing the gradual transition of perimenopause. Chemotherapy can also provoke a permanent damage in ovaries and induces menopause *per se* [1]. Women who experience an early menopause are more susceptible to certain health problems, such as osteoporosis and heart diseases, since they spend more time in their lives without the benefits of estrogens. POF can also be temporary (temporary menopause) induced by high levels of stress, excessive exercising and/or dieting, and by medications used to treat fibroids [2] and endometriosis [3]. However, as soon as women adopt a healthier life style or stop medication, the ovaries may resume normal production of hormones. Normally, menopausal transition or perimenopause starts around mid-to-late 40s and persists several years before the last menstrual period, normally for 4-5 years (Figure 1). Smoking and genetic background are two factors that can influence the timing of spontaneous menopause. Normally, smokers can reach menopause earlier than nonsmokers [4]. During perimenopause, levels of estrogen and progesterone start gradually to decline and menstrual periods become irregular. Since sex hormones are physiologically important to maintain the health and normal functioning of several organs, such as the heart, liver, brain, and bone, hormonal changes observed during this menopausal transition may induce several chronic medical conditions [5]. All women experience menopause, but different women may cope with different symptoms. The variation of menopause phenotypes around the world and in different ethnic groups suggests both cultural and genetic influences [6, 7]. Menstrual irregularities, vaginal atrophy, and vasomotor instability are the most frequent menopausal symptoms that have been directly related with the decreased levels of female sex hormones [8].

Menopause-associated vasomotor symptoms (also known as hot flashes) include spontaneous feeling of warmth, usually on face, neck, and chest and are usually associated with perspiration, palpitations, and anxiety, being variable in frequency, duration, and severity, and can be the cause for fatigue, difficulty concentrating, and memory lapses, symptoms that have also been observed during menopause transition. The cause for menopause-associated vasomotor symptoms is not completely understood, although some theories have been proposed [8, 9].

Vaginal atrophy is also a common symptom during menopause transition. Due to loss of estrogens, vagina lining may become thinner and dryer, and the pH also changes, making the vagina more susceptible to infections. Those alterations can affect sexual function and quality of life [10].

Others menopause-associated complications include increased cardiovascular risk (see below), osteoporosis [11] and body weight gain, which can all be a combination of changes in hormone levels and aging.

Increase in body weight is another characteristic associated with menopause. Although it is known that the metabolic rate decreases with aging, the increase in body weight and visceral adipose tissue accumulation after menopause have been associated with ovarian hormone withdrawal [12]. It has been shown that, in abdominal adipocytes, estrogen regulates the expression of lipoprotein lipase (LPL) and hormone-sensitive lipase (HSL) [13]. In hepatocytes, estrogen regulates the synthesis of structural apolipoproteins for very low-density lipoproteins (VLDLs) and high-density lipoproteins (HDLs) and decreases the synthesis of hepatic lipases [14]. By regulating lipidogenesis in adipocytes and hepatocytes, estrogen modulates lipid concentration in plasma. The withdrawal of estrogens during induced or natural menopause leads to several lipid metabolism disorders. For example, dyslipidemia was also observed in bilateral oophorectomized in women [15]. Abdominal accumulation of adipose tissue and associated dyslipidemia are important components of a group of metabolic irregularities strongly related with increased cardiovascular risk in the menopausal woman.

## 2. Cardiovascular Disease in Women during Menopause: The Role of Hormone Replacement Therapy

### 2.1. Clinical Data: What Do We Know?

Cardiovascular disease (CVD) is a multifactorial disease. Both bad lifestyle including inappropriate diet, sedentary life, smoking and drinking, and determined factors (e.g., aging, sex, genotype, and menopause) influence CVD [16, 17]. The impact of CVD on overall mortality in westernized countries is enormous, accounting for up to 30% of all deaths worldwide. The definition of CVD includes four major groups of diseases: coronary heart disease (CHD) disclosed by angina pectoris, myocardial infarction, heart failure, and coronary death, cerebrovascular disease such as stroke or transient ischemic attack, clinically evident peripheral artery disease, aortic atherosclerosis, and thoracic or abdominal aortic aneurysm. What is less known is that CVD is the leading cause of death in women, with more deaths than all other causes combined yearly [18]. Various studies showed a growing risk for CVD in menopausal women due to negative changes in metabolism and hemodynamic parameters [16]. According to the guidelines of the National Cholesterol Education Program (NCEP) [19], the American Heart Association (AHA), and the American College of Cardiology (ACC) [18, 20], evaluation of CVD risk factors in women must include a personal CHD history, age over 55, family history of premature CHD, diabetes mellitus, dyslipidemia, hypertension, personal history of peripheral artery disease, and smoking.

Guidelines for prevention of CVD in women were first published in 1999 by the American Heart Association (AHA) [21]. One consequence of such increased attention to gender-related health problems, is awareness of CVD as the leading cause of death among women has nearly doubled since 1997 [22]. The impact of menopause should be taken into account when discussing CVD, and this aspect has been the matter of debate [23].

Premenopausal women have a lower incidence of CVD when compared to men with the same age-range. Whereas CHD is sporadic in premenopausal women [24], the incidence of myocardial infarction increases with age in both sexes, but occurs later and after menopause [24]. Estrogen loss during menopause causes negative effects on metabolism and cardiovascular function [25], and the progression to menopause with the changes in estrogen levels decreases or cancels the women advantage versus men [26–29].

Postmenopausal women have a higher risk of coronary artery disease, atherosclerosis, and all causes of mortality [29]. A consequence of this gender-related trend is that the postmenopausal state is acknowledged as a risk factor for CHD, with a weight similar to that of male sex [30]. Furthermore, an early natural menopause appears to be associated with increased risk of CVD [31, 32], even in non-smokers.

Indeed, menopause is associated with increased total serum cholesterol, triglycerides, and fibrinogen, as well as with a decrease in high-density lipoprotein (HDL) cholesterol. A plausible explanation is that menopause is believed to be a result of fluctuations in hormonal status, primarily a deficiency in estrogen [33]. Whether other contributing factors may have a role on CVD after menopause, is less clear and difficult to demonstrate. The transition from premenopausal phase to menopause, for example, may induce a weight gain responsible for increased in blood pressure, total cholesterol, low-density lipoprotein (LDL), triglycerides, and fasting insulin [33]. What should be mentioned is that aging *per se* can be more important than menopause itself for a number of CHD risk factors. In the SWAN study (Study of Women's Health Across the Nation) [34], changes in traditional risk markers of CHD were evaluated in three different stages: before, within a year, and after the final menstrual period within a multiethnic group (African, American, Hispanic, Japanese, or Chinese and Caucasian women). Changes due to menopause were only represented by total cholesterol, low-density lipoprotein cholesterol, and apolipoprotein B. By contrast, chronological aging was responsible for changes in the other risk factors with a linear model. Many other potential factors might be also implicated in the sex differences in coronary heart disease [35]. The possibility that heart disease risk determines menopausal age rather than the inverse has already been proposed [36].

Oxidative stress plays a role in hypertension, hypercholesterolemia, diabetes, and promoting CVD [37]. The formation of free radicals leads to cellular oxidative stress with a contribution to the first step of endothelial damage and the progression to atherosclerotic lesion. The perpetuation of the process induces the final events of CVD, which appears to be linked to some oxidative stress biomarkers [38, 39]. Oxidative stress appears to be an emerging factor also in the pathophysiology of CVD in menopausal women. Studies have shown that during menopause the risk of CVD increases at the same time of a rise in oxidative status [40, 41].

It is still unclear if the type of menopause (surgical or natural) can have a role on cardiovascular risk. The Nurses' Health Study (1987) demonstrated that the risk of CHD was higher in patients undergoing bilateral oophorectomy compared with natural menopause. An estrogen-replacement therapy could prevent this effect [42]. In a later study, carotid artery intima-media thickness showed a positively association with years elapsed since menopause; however, according to this marker of subclinical atherosclerosis, women with natural menopause presented no difference compared with those who had surgical menopause [43]. Indeed, men with the common estrogen receptor alpha (ESR1) c.454-397CC genotype have a major risk of myocardial infarction, suggesting the potential linkage between estrogen receptors and CVD susceptibility. In this respect, a variation in estrogen receptor could clarify the contrasting results of hormone therapy on CVD susceptibility in women [44]. The apparent protective effect of hormone replacement therapy (HRT) has been a matter of debate for several years [45–47]. Prevention of CHD and osteoporosis in menopausal women was originally achieved by exogenous estrogen plus progestin, assuming a protective effect of estrogen on the heart. Additional effects included a protective effect on the bone and on colon cancer [48–52], despite increasing incidence of breast cancer [53, 54]. Two landmark studies, however, changed this view. The Women's Health Initiative (WHI) Estrogen plus Progestin (E+P) trial in 2002 showed no protection for CHD and confirmed the increased risk in breast cancer and thromboembolic disease [55].

Two years later the WHI Estrogen Alone trial confirmed the lack of effect on CHD while suggesting a trend for decreased breast cancer, with a rise in stroke and venous thromboembolic disease. A nonsignificant protective effect on CHD was seen in the younger women (ages 50 to 59) [56]. The public consequence was that hormone therapy was abandoned or was conducted with lower doses [57].

The possibility that CHD risk is lowered by earlier hormone therapy after menopause should also be considered, although results are not conclusive [58]. Whether hormone replacement therapy results in either increased or unchanged risk for stroke, is also a matter of debate [56]. Of note, recent guidelines do not identify estrogen therapy for the primary or secondary prevention of CHD [59, 60].

### 2.2. Animal Models: Helping to Define the Role of Estrogens

Although the WHI and the Heart and Estrogen/progestin Replacement Studies (HERS) showed no CVD protection resulting from HRT, several animal studies have suggested an important cardioprotective role for estrogens against heart failure [61], mediated by a genomic or a nongenomic estrogen-receptor-mediated signaling pathway (see [62] for a review).

Tumor necrosis factor-alpha (TNF-*α*) has been reported as an important factor during I/R injury and ischemia preconditioning. In a Langendorff-perfused rat heart model, estrogen reversed the deterioration of heart hemodynamics induced by TNF-*α* treatment [63]. Several evidences have been demonstrated that stromal cell-derived factor 1 (SDF-1) is increased in ischemic hearts and induced cardioprotection [64]. A higher expression of myocardial SDF-1 was observed in female rats in response to I/R and the increased myocardial SDF-1 production in female hearts was due to estrogen-estrogen Receptor *α* (ER*α*) interactions [65]. In C57BL/6J male mice, estrogen also induced cardioprotection after acute myocardial infarction through a decreased activity of matrix metalloproteinase-9 and increased Akt-Bcl-2 antiapoptotic signaling [66]. In a Langendorff isolated perfused rat heart model, estrogen increased the perfusion pressure and coronary resistance through activation of L-type calcium channels [67].

Estrogen-related receptor alpha (ERR*α*) is a transcription factor for some myocardial mitochondrial enzymes, essential to maintain cardiac energy reserves. A decrease in myocardial ERR*α*, regulated by the metabolic sensor AMP-activated protein kinase alpha 2 (AMPK*α*2), was recently reported during congestive heart failure [68]. Proteins from the intracellular lipin family are also involved in metabolism regulation. It was reported that lipin 1 is the principal protein of this family in myocardium and is also regulated by ERR*α* [69].

The lack of CVD protection observed during HRT has been proposed to be related with alterations in sex hormone synthesis and metabolism that can occur during aging, and can affect the hormone environment in postmenopausal women. Also age-related changes in vascular estrogen receptors (ERs) subtype, structure, expression, distribution, and the signaling pathway in the endothelium and vascular smooth muscle, preexisting CVD conditions, and structural changes in blood vessels architecture have been suggested as possible causes for the failure of HRT in CVD [70]. It also should be noticed that HRT is not only composed by estrogens, but also by a combination of estrogen and progesterone. A recent study demonstrated that a combination of 17-*α*-estradiol and medroxyprogesterone acetate aggravates chronic heart failure after experimental myocardial infarction, which can also explain the results from previous studies including WHI and HERS [71].

## 3. Cardiac Mitochondrial Fatty Acid Beta-Oxidation in Health and Disease: Where Does Menopause Stand?

The heart is one of the organs with the highest energy demand in the body, which is hardly surprising due to high energetic input required by the contractile apparatus. Although the heart is considered an omnivorous organ due to the fact that it can use several substrates for energy generation, including glucose, amino acids, lactate, and ketone bodies, fatty acids are the favored fuel for the cardiac muscle [72, 73]. In fact, the adult heart generates between 50–70% of its ATP from fatty acid beta-oxidation, which occurs mainly in mitochondria [72], possesses an elaborate system to import and process fatty acids of different lengths [72, 74]. In fact, in itself, mitochondrial function is one among different factors that impact the flux of fatty acid beta-oxidation. Others include the fatty acid supply itself, which is modulated among other factors by diet, competing substrates for the cardiac tissue, the energy demand and oxygen availability, and the regulation at a nuclear or allosteric level of enzymes which are involved in all steps of fatty acid uptake, esterification, and metabolism [72].

Fatty acids can be transported in the plasma as free fatty acids (FFAs) conjugated with albumin or as part of triacylglycerol (TAG) contained in chylomicrons or very-low density lipoproteins (VLDLs) [75, 76]. FFA concentration in the plasma is highly variable, depending not only on the diet, but also on the developmental state of the organism and if any pathology is present. For example, the amount of FFA in the plasma is known to greatly increase during myocardial infarction [77] and diabetes [78], which leads to an augmented cardiomyocyte FFA uptake and accumulation, since the concentration of FFA in the plasma is a major determinant for these two events [72]. Regardless of the mechanism underlying an acute or chronic accumulation of FFA in the plasma (reviewed in [72]), the end result of cardiomyocyte cytosolic accumulation of fatty acids can differ, depending on a wide range of factors.

The first step after entering the cardiomyocyte is conversion to CoA esters, through the action of fatty acyl CoA synthase (FACS). Fatty acid uptake by cells is made by membrane proteins with high affinity for fatty acids [79, 80], namely, the fatty acid translocase (FAT/CD36), the fatty acid binding protein (FABPpm) and a variety of fatty acid transport proteins (FATPs), as well as by simple diffusion of fatty acids through either the phospholipid bilayer or a pore or channel formed by one or more of the referred fatty acid transporter proteins [81]. Upon entering the cell, the rate of utilization is governed by a variety of factors, including malonyl-CoA, the ratio acetyl-CoA/CoA and the availability of other substrates, namely, glucose, lactate, and ketone bodies that can compete with free fatty acids as a source of acetyl-CoA [79]. Long-term regulation of uptake and utilization requires alterations in expression rates of genes encoding for fatty acid handling proteins [82]. Free fatty acids can also by themselves modulate the expression of such genes via nuclear transcription factors such as peroxisome proliferator-activated receptors (PPARs) [83].

**Figure 2:**
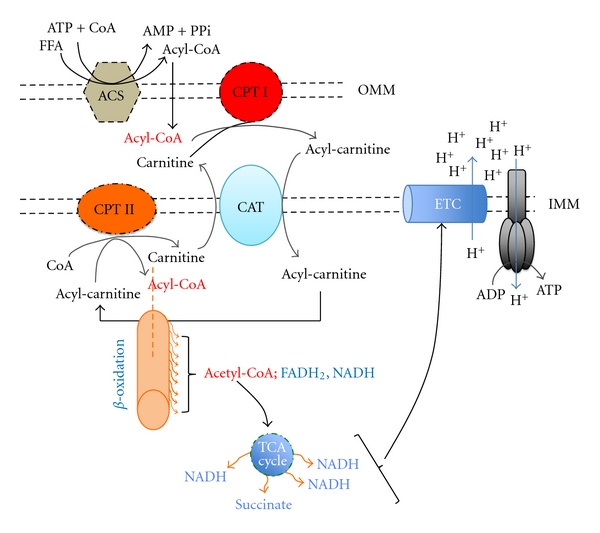
Transport of fatty acids from the cytoplasm to the mitochondrial matrix for oxidation. Following activation to acyl-CoA, CoA is exchanged for carnitine by carnitine palmityl transferase (CPT-I), which is then transported to the inside of the mitochondria where a reversal exchange takes place through the action of carnitine acylcarnitine translocase (CPT-II), and beta-oxidation machinery initiates its activity, producing reducing equivalents that feed the electron transport chain. More details are available in the text. CAT: Carnitine Acylcarnitine Translocase, FFA: free fatty acid, ACS: Acyl-coA synthase, ETC: electron transport chain, IMM: inner mitochondrial membrane, OMM: outer mitochondrial membrane, coA: coenzyme A, ATP: adenosine triphosphate, ADP: adenosine diphosphate, and AMP: adenosine monophosphate.

Mitochondrial beta-oxidation of long-chain fatty acids starts with its association with CoA, forming acyl-CoA esters that are transported into mitochondria by carnitine palmitoyl transferase I (CPT-I). Beta-oxidation produces in each round one NADH, one FADH_2_ (as part of an enzymatic complex), and one acetyl-CoA, which is further oxidized in the Krebs cycle to CO_2_, with the concomitant further generation of three NADH, reduced FAD co-factor in succinate dehydrogenase complex, and one GTP. NADH, via NADH dehydrogenase, and succinate dehydrogenase deliver electrons to the remaining electron transport chain complexes which contribute to the generation of a proton gradient used to synthesize ATP (Figure 2). Throughout this whole process, several regulation mechanisms can operate, starting with the transport of the acyl chain to the mitochondrial matrix and ending at the accumulation of end products of the oxidation process, namely, reducing equivalents and ultimately ATP levels. The transport process is considered a major player in the control of the flux through beta-oxidation [84], mostly in intact muscle, since levels of malonyl-CoA are kept considerably high. With this type of control, it is possible for the tissues to rapidly adapt to different metabolic demands, such as in muscles [84]. An inhibition of fatty acid beta-oxidation, which as mentioned can occur at several stages, will ultimately result in free fatty acid intracellular accumulation which subsequently will be responsible for poor removal of fatty acids from plasma in any of their forms of transportation. In fact, a possible role has been attributed to female sex hormones in the development of fatty liver pregnancy on the basis of their effect in the reduction of mitochondrial fatty acid oxidation [85] and in regulating cellular energy balance *in vivo* by regulating the expression of the medium chain acyl coenzyme A dehydrogenase (MCAD) gene [86].

Besides mitochondrial oxidation, long-chain fatty acyl coA can also be used for the synthesis of intermediates, including TAG, diacylglycerol (DAG), and ceramide [72, 87]. Under normal intracellular concentrations, these intermediates are stored and/or channeled to different biosynthetic pathways, including biomembrane synthesis. If alterations in normal fatty acid homeostasis occur, which can originate from excessive plasma FFA content or from enhanced FACS expression and/or activity, long-chain fatty acyl coA derivatives can accumulate in cells. Depending on the tissue, accumulation of some of these intermediates can have distinct effects. For example, it is known that excessive accumulation of TAG in nonadipocyte tissues can result in different negative outcomes including impaired insulin signaling in the liver and skeletal muscle [88] and apoptosis and other metabolic disturbances in the heart [87, 89, 90]. DAG has also been determined to cause similar effects in the same tissues [88], including increased insulin resistance observed in a model of rodent high-fat diet [91]. It is interesting to note that both increases in TAG and ceramide intracardiac content did not correlate with the increased insulin resistance [91].

Ceramide, by its turn, has been demonstrated in different biological models to increase apoptotic signaling in several tissues [92–94], although evidence is scarcer for the heart [95]. It is interesting to note that ceramide derivatives have been involved in the triggering of the mitochondrial permeability transition pore (MPT pore) and outer-membrane permeabilization [96, 97], conditions closely linked with mitochondrial dysfunction and cell death [98]. In opposition, long-chain ceramide species have been shown to inhibit the MPT pore [99]. The discrepancy of results regarding ceramide implicates this lipid species in the control of mitochondrial cell death pathways.

From the short description above, it is clear that a balance between FFA cell uptake and metabolism must be reached in order to avoid the accumulation of undesired fatty acid metabolites. Also, increased reliance of fatty acids as fuel for cardiac cells has undesired effects, one of them being decreased ATP synthesis, resulting from increased ATP hydrolysis for noncontractile purposes, increased mitochondrial uncoupling due to increased activity/expression of uncoupling proteins and greater proton futile cycling, creating the so-called oxygen wasting and resulting in several physiological complications [100–102]. Interestingly, inhibition of fatty acid metabolism is proposed to be beneficial for some forms of heart failure [103].

**Figure 3:**
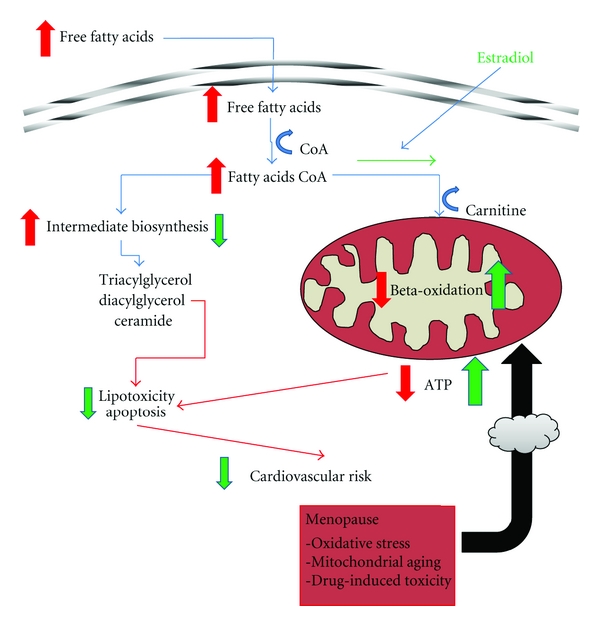
General scheme of the hypothesis raised by the present paper. It is proposed that menopause, as a condition natural to the normal aging process, is accompanied by specific mitochondrial alterations (bottom red box, arrow with a dark cloud) which decrease their ability to cope with an increased flux of long-chain fatty acyl CoA, resulting from augmented plasma levels. Inability to process fatty acyl CoA may result in accumulation of fatty acid intermediates including tri- and diacylglycerol, as well as ceramide, which causes myocardial lipotoxicity and may even result into activation of apoptotic signaling. The cardiovascular risk increases under these circumstances, which is fueled by other coexisting pathological conditions or by pharmacological interventions that present toxicity to the cardiovascular system. Estradiol (represented by green arrows) has been proposed to increase fatty acid oxidation by mitochondria, decreasing the flux through other biosynthetic pathways, preventing the potential accumulation of deleterious metabolites and increasing fatty acid-derived mitochondrial ATP production.

The important question is now where the menopausal heart stands. As described above, menopause is a normal consequence of the aging process in women and is accompanied of important physiological and biochemical alterations. There are several evidences in the literature that the content in FFA in the plasma tends to increase during menopause. One particular study performed with 4-vinylcyclohexene-diepoxide- (VCD-) treated rats indicated that progressive loss of ovarian function induced by VCD results in an increase of plasma FFA, which initiated several alterations leading to the development of the metabolic syndrome [104]. This important piece of evidence mimics what is observed in the menopausal women, where an increase in circulating FFA was measured [105]. It is also known that women experience a characteristic increase in circulating lipids at the time of the final menstruation period [34], although it is difficult to evaluate the component resulting from hormonal alterations and what is the result of the normal aging process [34, 106]. The increased FFA was partly reverted by hormone-replacement therapy, showing that, at least in part, it is a hormone-dependent effect [105]. The role of estrogens in fatty acid metabolism is well described and involves different mechanisms [107–109]. One important effect is that estradiol promotes the channeling of FFA toward oxidation and away from triglyceride storage (Figure 3) by upregulating the expression of peroxisome proliferation activator receptor delta and its targets and also by directly and rapidly activating AMP-activated protein kinase (AMPK). AMPK acts as a fuel sensor that increased fatty acid beta-oxidation during higher metabolic demands [110].

The data, although still scarce and largely spread out, indicates that during menopause, fatty acid metabolism is altered. The decrease in estradiol levels may result in decreased fatty acid oxidation and increased accumulation in the adipose tissue, with hormone replacement therapies recovering the pre-menopausal fatty acid status quo. But is this so straightforward? Maybe not, one important player in fatty acid metabolism is, as described, the mitochondrion. A proper channeling of fatty acyl-CoA and subsequent beta-oxidation is necessary for the energy-generating process. It is clear that a failure of mitochondrial bioenergetics causes an unbalance in fatty acid metabolism, which may result in the accumulation of fatty acyl-CoA esters in the cytosol of cardiomyocytes. This phenomenon could result in a larger channeling of fatty acyl-CoA esters to the synthesis of the intermediates described above, including TAG, DAG, and ceramide. It is interesting to recapitulate here that ceramide has been involved in the induction of apoptosis in a variety of biological models [92–94]. Although the relationship between increased ceramide intracellular levels in the menopausal heart and increased apoptotic signaling is still to be determined, several endpoints for increased cardiac Fas-dependent and mitochondrial-dependent apoptosis were identified in the hearts of bilateral ovariectomized Wistar rats [111, 112]. A logical question would be if there is a possible relationship between intracellular lipid metabolism alterations resulting from ovariectomy and enhanced apoptotic signaling in the heart.

Decreased fatty acid oxidation by mitochondria occurs in a variety of situations, ranging from xenobiotic-induced toxicity to several pathologies. There are many fatty acid oxidation disorders identified in humans, and which affect organs as different as muscle [113] and brain [114], which result in altered fat deposition and mitochondrial beta-oxidation. Defects are commonly present in the mitochondrial machinery that shuttles long-chain fatty acid metabolites to mitochondria, resulting in decreased beta-oxidation [113]. Several xenobiotics also alter fatty acid metabolism in different organs [115], examples are fluorochemicals [116] and the antibiotic tetracycline [117] in the liver. As for the heart, it is now becoming increasingly recognized that alterations in fatty acid uptake and/or beta-oxidation can result in the so-called fatty heart, a largely unrecognized entity for a long time, and which, as described has important cardiovascular complications [89, 118]. This subject will deserve more attention in the future.

It has been proposed that mitochondrial function in the heart decreases with the progression of aging. Alterations include loss or oxidation of cardiolipin, a tetra-acyl phospholipid involved in the activity of many oxidative phosphorylation enzymes including complex I [119–121]. This presents a clear determinant of loss of mitochondrial function and also represents a phenotype of mitochondrial membrane aging which impacts both the bioenergetics and several signaling pathways to and from mitochondria.

It is also known that aging-dependent cardiac mitochondrial effects are more specific to interfibrillar mitochondria, which is the subpopulation responsible for the majority of energy supply to the myocardium [122, 123]. Such alterations include decrease respiratory complex activity and increased oxidative stress, while a decreased capacity for beta-oxidation has also been demonstrated in an animal model for aging due to alterations in carnitine palmitoyltransferase I which were suspected to originate from a decrease in cardiolipin content [123]. Mitochondrial “power” in the heart is thus affected with aging [124], which is further illustrated by a decrease in the nuclear control of mitochondrial biogenesis and function [125] and by increased mtDNA deletions frequency found in the aged heart [126]. 

Adding to mitochondrial aging, *per se*, one has to have in mind that other factors may be operating in the menopausal woman that can contribute to altered mitochondrial function and result in disrupted fatty acid metabolism. For example, the incidence of diabetes, and obesity increases during menopause [127], which also contributes to accelerate mitochondrial dysfunction [128–130]. By its turn, the menopausal woman may be under treatment with different medications which may also affect the bioenergetic efficacy of cardiac mitochondria [131, 132], especially if other conditions occur at the same time.

To summarize, ageing results into a progressive degradation of mitochondrial capacity in the heart, which, in combination with hormonal alterations resulting from menopause and its associated alterations in lipid profile, may result into a progressive decrease in lipid oxidation in mitochondria and increased lipid storage in adipocytes and formation of fatty acyl intermediates in the cytosol of cardiomyocytes (Figure 3). The development of insulin resistance, diabetes and obesity can be several faces of the same coin, the increased lipotoxicity in the cardiomyocyte of the menopausal woman. This is a clear avenue for research that still is largely unexplored and deserves attention since menopause is a condition that affects an increasingly number of women, as the general population is progressively aging.

If the hypothesis put together in this paper is correct, then prophylactic measures that improve mitochondrial capacity in menopausal women would contribute to decrease cardiovascular risk. In fact, besides hormone replacement therapy, which replenishes estrogens and reequilibrates lipid homeostasis, other cotherapies may help improve the lipid profile in the menopausal woman through different mechanisms. For example, endurance exercise has been demonstrated to increase mitochondrial capacity in the heart [133, 134]. In a menopausal setting, twelve weeks of endurance exercise have been demonstrated to provide some benefits in increasing lipid oxidation, besides improving other cardiorespiratory parameters [135, 136]. Carnitine, which is essential to long-chain fatty acid beta-oxidation, has been shown to recover some of skeletal muscle function and inhibit alterations in ovariectomized rats [137]. Nevertheless, to the best of our knowledge, no work on the impact of carnitine on lipid profile and oxidation in the menopausal heart has been provided.

Cardiac oxidative stress after ovariectomy has also been observed in animal models [138] although evidence for increased oxidative stress in the cardiovascular system is scarce. Estrogens *per se* act as antioxidants, although it is still unclear if estrogen supplementation during menopause is completely without risks for the cardiovascular system [139, 140]. Also, it is unclear so far if antioxidant supplementations would improve mitochondrial fitness in menopausal women. Finally, an interesting alternative was proposed by Zern et al. [141]. Lyophilized grape powder was given to a group of postmenopausal women for 4 weeks. The powder was enriched in phytochemicals such as flavans, anthocyanins, quercetin, myricetin, kaempferol, and resveratrol. The results showed alterations in lipoprotein metabolism, oxidative stress, and inflammatory markers, which were all decreased in the treated group. Although the heart was not specifically targeted in the study, the results may suggest a positive impact in this organ as well. Interestingly, resveratrol is considered an activator of mitochondrial biogenesis in different model systems, acting through sirtuin-1-dependent and independent mechanisms [142–144]. The future will tell if this is a trail worth exploring.

## 4. Concluding Remarks

Although there are many loose ends in the story, it appears logical to consider that progressive deterioration of mitochondrial function in the aging woman with menopause contributes to the metabolic alterations observed in the heart, including a decreased capacity for lipid oxidation. A decreased mitochondrial flux of fatty acid beta-oxidation, can result in most cases in the accumulation of toxic intermediates in the cytosol and also of nonmetabolized fatty acids in mitochondria, which leads to further deterioration of mitochondrial function and progressive metabolic changes that can increase cardiovascular risk. Not only this line of thought needs to be demonstrated in animal models and humans, but if true, pharmacological, or nonpharmacological strategies must be devised to counteract this metabolic remodeling.
